# Chronic Kidney Disease of Unknown Origin: A Mysterious Epidemic

**DOI:** 10.7759/cureus.17132

**Published:** 2021-08-12

**Authors:** Gokul Paidi, Anuruddhika I Iroshani Jayarathna, Divya Bala Anthony Manisha R Salibindla, Jashvini Amirthalingam, Katarzyna Karpinska-Leydier, Khadija Alshowaikh, Huseyin Ekin Ergin

**Affiliations:** 1 Internal Medicine, California Institute of Behavioral Neurosciences & Psychology, Fairfield, USA; 2 Neurology, California Institute of Behavioral Neurosciences & Psychology, Fairfield, USA; 3 General Medicine, California Institute of Behavioral Neurosciences & Psychology, Fairfield, USA; 4 Obstetrics and Gynecology, California Institute of Behavioral Neurosciences & Psychology, Fairfield, USA; 5 General Practice, California Institute of Behavioral Neurosciences & Psychology, Fairfield, USA

**Keywords:** chronic kidney disease (ckd), uddanam nephropathy, balkan endemic nephropathy, tondaimandalam nephropathy, unknown etiology, sri lanka nephropathy, mesoamerican nephropathy

## Abstract

Amongst the many threats to health in our world, the most challenging ones are the ones with unknown causes. There is a mysterious epidemic of kidney disease called chronic kidney disease of unknown etiology (CKDu) that is occurring in many parts of the world. Unrelated to known risk factors such as diabetes and hypertension, CKDu mostly affects the young and middle-aged, with slight preponderance in males. It mostly occurs in people living in rural areas, especially working in agricultural jobs. Worldwide, the number of people with chronic kidney disease, and those who need dialysis and renal replacement, is increasing every year as compared to other chronic conditions like diabetes and AIDS. It’s not just alarming but a great challenge to healthcare systems across the world, especially in resource-poor countries. CKDu has become a silent killer for most patients. The occurrence of end-stage renal disease (ESRD) in CKDu can be catastrophic for individuals, especially in countries with limited medical facilities, causing a significant socio-economic burden. Even within these economically developing nations, people affected by CKDu usually are from the most vulnerable and underserved populations. As a definitive etiology has not been postulated for CKDu to date, this comprehensive review was undertaken to throw light on the poorly understood epidemiologic risk factors and the course of the disease.

## Introduction and background

Chronic kidney disease (CKD) could be a key determinant of unfavorable health consequences and is regarded as an independent risk factor for cardiovascular disease events. There has been a dramatic increase in the global burden of CKD over the last two decades [1]. Globally, the prevalence was estimated to be 147.6 million in 1990 and there was an increase to 275.9 million in 2016. Over the last two decades, there has been a doubling of crude mortality rate from approximately 0.59 million to 1.2 million [2]. The prevalence of CKD is disproportionately high in lower and middle-income economically developing countries as compared to high-income developed countries (>15% higher) [3]. Although the primary cause of CKD varies across countries, hypertension and diabetes are the most common causes [4]. However, there has been a drastic increase in the incidence and prevalence of CKD of unknown etiology (CKDu) across different geographical regions, without any known risk factors [5-7].

Other terms utilized include 'CKD of nontraditional etiology', 'constant interstitial nephritis in agricultural communities', and 'kidney illness of unknown reason in horticultural workers'. Since it is commonly encountered in low-income, tropical/subtropical areas, rural and middle-aged men in agricultural and similar work requiring hard manual labor [8], CKDu has gained interest among researchers and clinicians in recent times.

Not surprisingly, there is a shortage of data on CKDu. However, data from the Indian CKD Registry revealed that CKDu is the second-most common underlying cause of CKD (16.0%) after diabetic nephropathy (31.3%) [9]. CKDu has a multifactorial etiology of different environmental and occupational exposures, such as heat stress, dehydration, agrochemicals (pesticides, herbicides, fertilizers), heavy metals (cadmium, lead, arsenic, etc.), water sources, and infections. It is life-threatening due to late recognition and rapid disease progression. Early screening of etiological risk factors for CKDu is essential to reduce the mortality and morbidity due to CKD. Various risk factors associated with CKDu are arsenic and cadmium in water, dehydration, extreme physical exertion, heat stress, agrochemical exposure, traditional nephrotoxic drugs like aristolochic acid, smoking, alcohol made with local traditional methods, infections, snake bites, and family history of CKD.

In Sri Lanka, CKDu was first reported in 1994 [6]. In 2017 and 2018, the Sri Lankan Society of Nephrology rectified the epidemiologic term of CKDu [10]. Despite this definition, there is a lack of consensus on criteria for diagnosis outside of kidney biopsy, with characteristic environmental and lifestyle exposures hypothesized to play an important role in disease identification and diagnosis [11]. In histopathology, the primary feature in CKDu is fibrosis in kidney tubules and interstitium with glomerular and vascular damage in different stages of evolution.

## Review

Epidemiology

**Figure 1:**
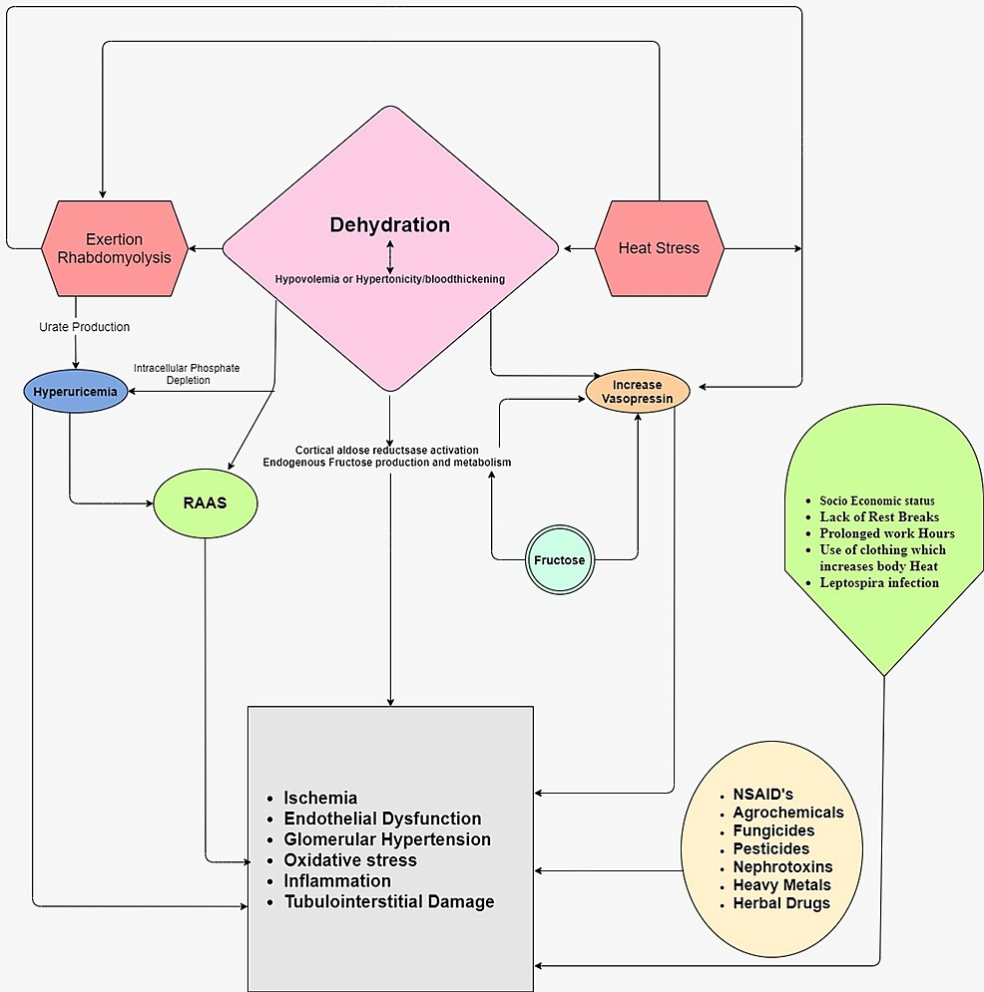
Pathogenesis of chronic kidney disease of unknown origin (CKDu) RAAS: Renin-angiotensin-aldosterone system; CKD: Chronic kidney disease; NSAIDs: Nonsteroidal anti-inflammatory drugs

There had been evidence of various outbreaks of CKDu in the last two decades like the Balkan endemic nephropathy (BEN) in the Balkan States, Itai Itai nephropathy caused by cadmium-contaminated rice intake in Japan, aristolochic acid nephropathy (AAN) among users of herbal medicine in China, Taiwan, and other countries, Uddanam endemic nephropathy (UEN) in India, mesoamerican nephropathy (MeN) in Central America, and Sri Lanka nephropathy [12-15]. Recently, there have been claims to rename CKDu as chronic interstitial nephritis in agricultural communities (CINAC) [16,17]. All these share common features that interact with each other as shown in Figure 1.

Balkan Endemic Nephropathy 

First reported in 1956 in Bulgaria, BEN is a chronic tubule-interstitial disease that is slowly progressive and has a familial etiology. It is associated with the atypia of the urothelium, which sometimes can result in cancers of the renal pelvis and urethra [18]. It usually occurs in the fifth decade of life and renal failure usually is reported in the sixth and seventh decades of life. In 1957, patients in Yugoslavia (Serbia) presented with similar clinical signs and since then many cases have been reported. Most of the reported cases were found in the Balkan peninsula and regions of southeast Europe. To be specific, it occurs among those living at the convergence of river Danube where there is high humidity and heavy rainfall [19]. The approximate prevalence of BEN in those regions ranges between 0.5-4.4% [20]. However, the prevalence could be as high as 20% if the screening of high-risk populations is done since nearly all affected patients are farmers. It is noteworthy to mention that nearly 10% of patients in Bosnia with BEN present during the end-stage kidney failure (ESKD) stage [21]. The etiological factors, which were divided into exogenous and endogenous factors are shown in Table 1.

**Table 1:** Etiology of Balkan endemic nephropathy (BEN) LCAT: Lecithin-cholesterol acyltransferase

Exogenous factors	Endogenous factors
Lead intoxication	Genetic predisposition
Metals and metalloids	LCAT enzyme deficiency
Intoxication with *Aristolochia* *clematitis*	Genetic polymorphism
Ochratoxin A	Chromosomal aberrations
Pliocene lignite	Viral disease
Lecithin cholesterol acyltransferase and organic substances from coal	Immunological factors

Literature has reported genetic predisposition to be the primary cause for BEN. There are strong arguments that environmental etiology also plays an important role in BEN such as chronic poisoning with aristolochic acids. The fact that it has been difficult to decipher the etiology of BEN for almost 50 years makes it valid to accept the argument that BEN may be caused due to the interplay of multiple etiologies. The clinical features of BEN usually range from non-specific symptoms to weakness and fatigue along with lumbar pain and discoloration of the palms and soles (copper-brown discoloration). At an advanced stage, it presents with anemia and loss of renal function leading to ESRD. Intermittent proteinuria, which is present in the earlier stage, becomes permanent in the uremic phase of the disease [22-24].

Itai-Itai Disease

Itai-Itai disease ('it hurts-it hurts' disease) was officially recognized in 1968 and was caused by activities related to industrialization. This chronic kidney disease mainly targets women living in rice farming regions near the polluted Jinzu river, which is high in cadmium and other heavy metals in Toyama, Japan. It has been acknowledged since the 1950s by the effort of residents and Dr.Hagino who was practicing general medicine in a clinic in the polluted river areas [25,26]. It's a kind of acquired Fanconi syndrome, portrayed by renal tubular dysfunction and osteomalacia, induced by chronic cadmium exposure through contaminated rice and drinking water [27,28]. These immunological adjustments of CKD might be seen in patients with Itai-Itai illness, causing infections, for example, pneumonia and gastrointestinal (GI) inflammation hence giving rise to high mortality [29].

Aristolochic Acid Nephropathy

**Figure 2:**
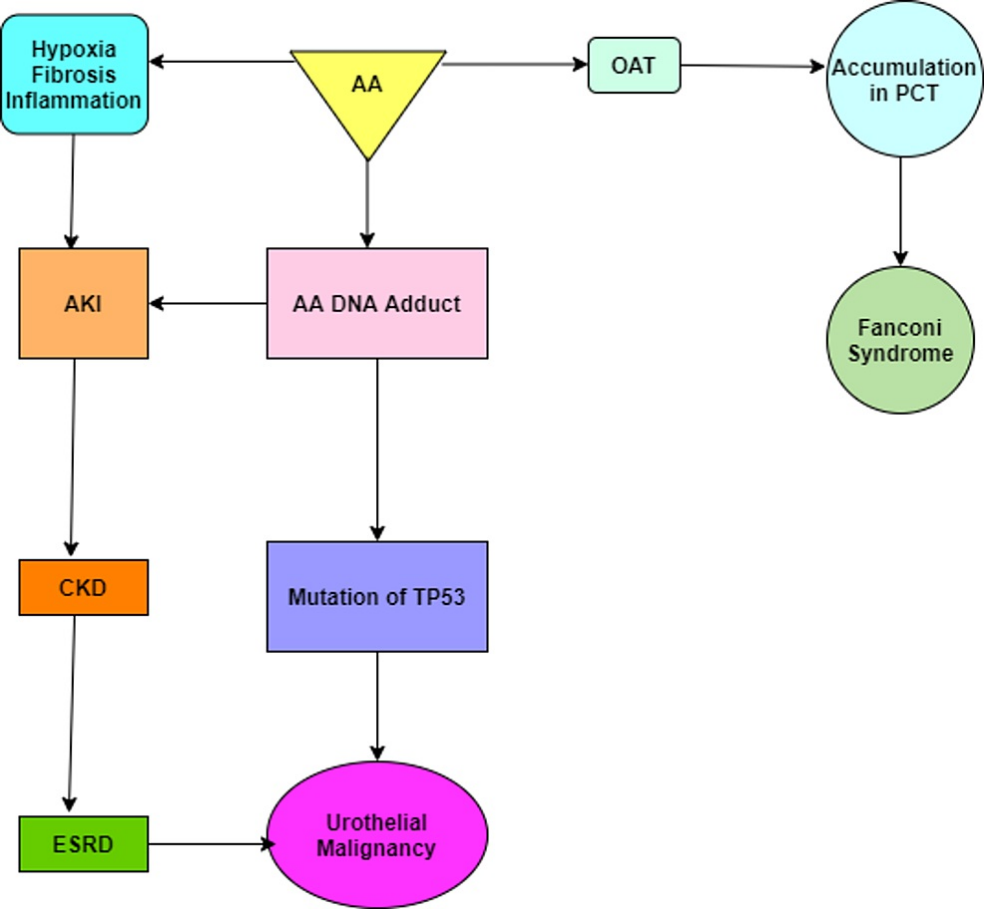
Pathogenesis of Aristolochic acid nephropathy (AAN) AA: Aristolochic acid; AKI: Acute kidney injury; OAT: Organic anion transporter; CKD: Chronic kidney disease; PCT: Proximal convoluted tubule; ESRD: End-stage renal disease; TP53: Tumor protein P53

A quickly progressive interstitial nephritis prompting ESRD and urothelial malignancy, AAN was first revealed in Belgium in a class of patients who had ingested weight loss pills containing powdered root concentrates of Chinese spices [30-32]. In most Asian countries, up to 70% of people rely on conventional traditional medicines, which are made with different plant and animal products and there is a high risk of the disease in these countries due to the frequent use of aristolochic species [33,34]. AAN has predominantly raised serum creatinine, huge pallor, and histopathologic changes showing hypocellular interstitial infiltrates with serious fibrosis. Proceeding toward ESRD is fast, with most patients having a persistent kidney infection for under two years. Moreover, AAN is related to a 40-45% predominance of urothelial carcinomas [35]. The pathogenesis of AAN is shown in Figure 2.

Aristolochic acid exposure is further associated with BEN; nevertheless, the disease course to kidney failure is significantly longer in AAN.

Mesoamerican Nephropathy

**Figure 3:**
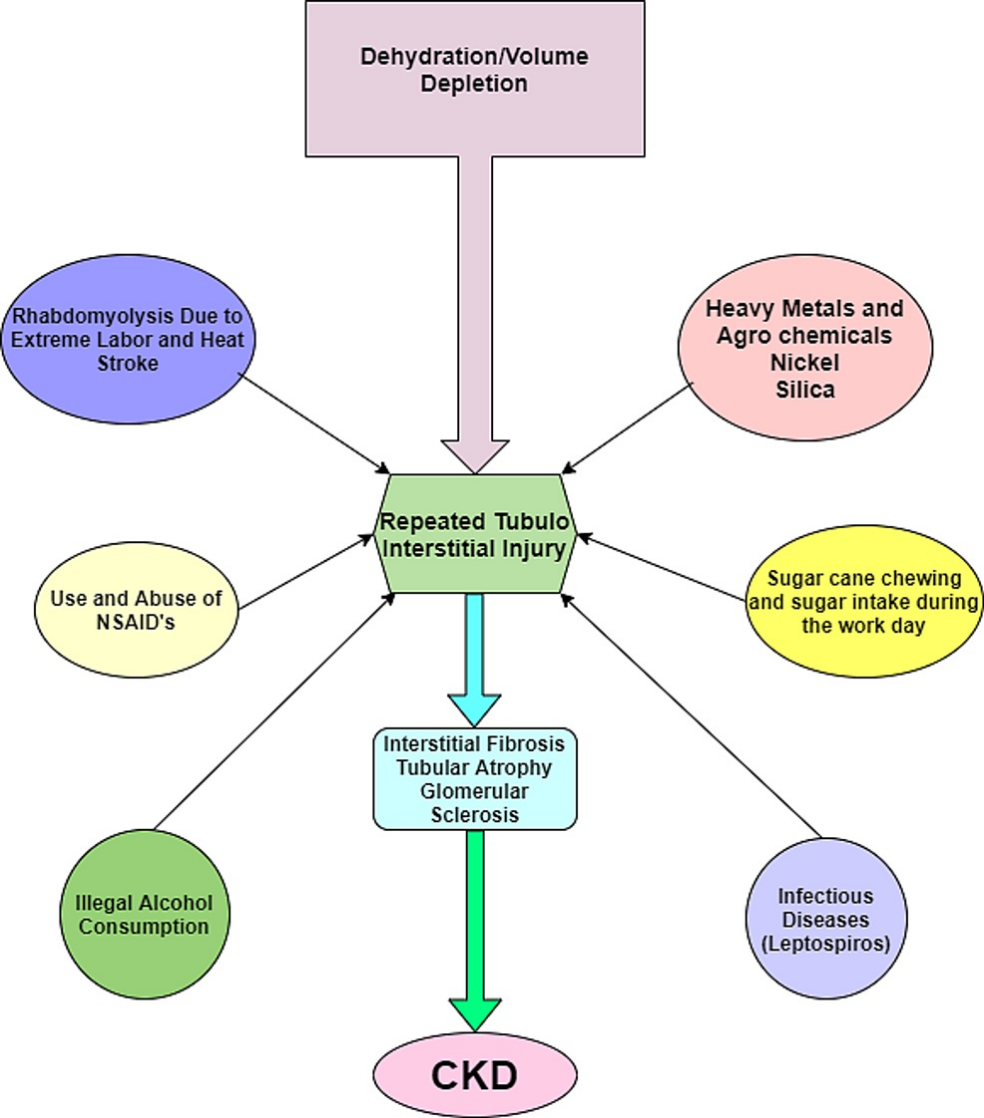
Pathogenesis of Mesoamerican Nephropathy (MeN) CKD: Chronic kidney disease; NSAIDs: Non-steroidal anti-inflammatory drugs

MeN or CKD of nontraditional reason [36] corresponds to CKD that presents in rural farm laborers, especially those working in sugarcane farms in Central America. [37,38]. The risk factors and etiology of MeN are unclear and debatable, and current evidence is limited. It is thought to be associated with poor living and occupational conditions with repeated episodes of heat stress, hypovolemia, loss of mineral dehydration, and prolonged working hours with lack of breaks use of clothing, which increases body heat [39-41]. Recently, nickel has shown a high association with the disease. Factors leading to MeN are shown in Figure 3.

 

Clinically, MeN manifests as a tubulointerstitial disease. Urinalysis shows low-range or no proteinuria, chemistry shows low serum potassium and sodium, and large degrees of uric acid, yet no hypertension. Histopathological features of the disease incorporate tubular atrophy, interstitial fibrosis, and global glomerulosclerosis.

Sri Lanka Nephropathy

First reported in the 1990s in Sri Lanka in the North Central Province, it is also called chronic agricultural nephropathy or CKD of multifactorial origin [42-43]. The reported prevalence of 8-21% is felt to be underreported due to the lack of sufficient data of epidemiologic studies on the disease [16]. The available literature has found that the disease is common among males and the rural population [44-46]. Although the similarities between endemics of CKDu in Central America and Sri Lanka are close, we lake proof in the matter of renal morphology. Sri Lankan nephropathy etiology has not yet been postulated but exposure to pesticides, fertilizers, heavy metals, hardness of water, and certain infections is suspected to play a role [47-50]. Urine analysis has pointed toward heavy metal exposure as a possible cause but with limited validity [45,51]. The renal morphology of Sri Lanka nephropathy is frequently portrayed as tubulointerstitial nephritis/tubulointerstitial disease; however, huge glomerular lesions like total glomerulosclerosis, indications of glomerular ischemia, and inconsistent findings of focal glomerulosclerosis have additionally been accounted for [44,45,52-54].

Uddanam Endemic Nephropathy

UEN was first reported in 1990 in the Uddanam region located in the Srikakulam district of Andhra Pradesh, India [55]. As of 2015, around 34,000 people had kidney diseases in Uddanam [13,56]. The disease is known to disproportionately affect farmers and agricultural workers. Though it has been over thirty years since the first cases were reported, the cause of UEN is yet to be established. A study by Harvard University had reported that high levels of silica and heavy metals in water, prolonged dehydration, heat stress nephropathy, use of non-steroidal anti-inflammatory drugs (NSAIDs), gene mutations, and high use of pesticides are the probable cause of the condition. In histopathology, glomeruli were normal without any increase in cellularity, segmental lesions, or crescents. A diverse degree of ischemic changes and glomerular inactivity in addition to the atrophy of renal tubules and fibrosis of interstitium was seen. The inflammation was predominantly lymphomononuclear and around the atrophic tubules [57].

Tondaimandalam Nephropathy

A new and high prevalence of CKDu was noted in Tondaimandalam, in the southeastern coastal districts of Tamil Nadu and Puducherry, India, similar to the Uddanam region. The condition was commonly noted among the rice paddy, sugarcane, and peanut farming laborers engaged in herding animals and construction work. The clinical features include asymptomatic earlier stages, low-grade or absent proteinuria, and bilateral small kidneys. However, substantial data on the same is scarce since it is a new entity [58]. The epidemiological classification, etiology, and histopathological findings of the various CKDus are presented in Table 2.

 

**Table 2:** Clinico-epidemiological features of major globally reported chronic kidney disease of unknown origin (CKDu) LCAT: Lecithin-cholesterol acyltransferase

Chronic Kidney Disease of unknown origin (CKDu)	Geographical region	Occupation	Etiology	Histopathology
Balkan endemic nephropathy (BEN)	Southeastern Europe (Balkans): Serbia, Bosnia and Herzegovina, Croatia, Romania, and Bulgaria.	Farmers	Exogenous: Lead intoxication, Metals and metalloids, Intoxication with *Aristolochia* *clematitis*, Ochratoxin A and Pliocene lignite Endogenous: Genetic predisposition, LCAT enzyme deficiency, Genetic polymorphism, Chromosomal aberrations, Viral disease and Immunological factors Miscellaneous: Lecithin cholesterol acyltransferase and organic substances from coal	Hyalinization of the glomeruli, predominantly in the external part of the cortex, severe vascular changes, interstitial fibrosis, and scant inflammatory cell infiltrate. The renal pelvis and ureters revealed multiple urothelial papillomas and atypical urothelial hyperplasia
Itai-Itai disease	Japan	Farmers	Chronic cadmium exposure through contaminated rice and drinking water	Tubulopathy of proximal tubules resulting in thinning of renal cortex and decrease in kidney weight
Aristolochic acid nephropathy	Asian countries mainly China	-	Traditional medicine with Aristolochia species (Chinese herb)	hypocellular interstitial infiltrate with severe fibrosis and urothelial carcinomas
Mesoamerican nephropathy	Coastal regions of central America including Nicaragua and El Salvador mainly and to some extent Costa Rica and Guatemala	Agricultural workers	NSAIDs, heavy metals and agrochemical exposure (inconsistent), rhabdomyolysis due to extreme labor and heat stroke, infectious diseases (leptospirosis)	Chronic tubulointerstitial disease with secondary glomerular and vascular damage; occasional global glomerulosclerosis from possible glomerular ischemia
Sri Lanka nephropathy	North Central Province of Sri Lanka	Agricultural workers	Chena farming (vegetable and other crops)	Chronic tubulointerstitial fibrosis with nonspecific interstitial inflammation; rare glomerular collapse, and sclerosis with fibrous intimal thickening and arteriolar hyalinosis
Uddanam endemic nephropathy	India (Andhra Pradesh)	Cashew nut, coconut, and rice farming	High levels of silica and heavy metals in water, prolonged dehydration, heat stress nephropathy, use of non-steroidal anti-inflammatory drugs (NSAIDs), gene mutations, and high use of pesticides	Tubular atrophy and interstitial fibrosis mainly with secondary glomerular and vascular changes
Tondaimandalam Nephropathy	India (northern coastal districts of Tamil Nadu and Pondicherry)	Rice paddy, sugarcane, peanut farming	Not studied	Not studied

## Conclusions

Diagnosis and treatment of CKDu is a relatively new phenomenon in areas where farming has been prevalent for centuries. This review was conducted mainly to find the similarities and differences between the CKDu in various geographical regions and different nations across the world. Although heat stress and dehydration are presently the largest research focus in Latin America, contamination of drinking water is a primary cause for CKDu in Asia with less emphasis on heat stress and dehydration. Apart from these variations, similarities in disease patterns include late presentation, prolonged non-symptomatic stage, non-glomerular proteinuria, and the absence of hypertension in the beginning stages of the disease leading to a decrease in glomerular filtration rate features that are typical of chronic tubulointerstitial disease. Moreover, they commonly affect rural farmers in tropical countries and are undetectable until the final stage when kidney failure is inevitable, and it becomes a burden for patients and their families, both financially and emotionally, to obtain dialysis. Renal replacement therapy is non-affordable and limited. Research studies to date that aim to pinpoint risk factors associated with CKDu are varied and limited in geographic scope. Without conducting studies that look at all possible etiologies across regions using a standardized approach, it is difficult to arrive at a standard management guideline and improved survival among these patients. Research should focus on developing specific biomarkers to detect the CKDu in the early stages of the disease so measures can be taken to halt the progression to ESRD. Region-specific research labs should be established in the affected areas across the world and should conduct more comprehensive research in collaboration with other regional labs by sharing information for public service and research purposes. Surveillance and standardized disease registries and monitoring systems for cases of CKD and CKDu are important. Strengthening national environmental toxicology and all epidemiologic networks by supporting open transparent information exchange is needed. Since CKDu is mostly occurring in resource-poor countries, the WHO should declare it a global epidemic and allocate more funding to these countries for research.
